# Plasma Rich in Growth Factors Enhances Cell Survival after in Situ Retinal Degeneration

**DOI:** 10.3390/ijms21207442

**Published:** 2020-10-09

**Authors:** Carlota Suárez-Barrio, Susana del Olmo-Aguado, Eva García-Pérez, Enol Artime, María de la Fuente, Francisco Muruzabal, Eduardo Anitua, Begoña Baamonde-Arbaiza, Luis Fernández-Vega, Jesús Merayo-Lloves

**Affiliations:** 1 Instituto Universitario Fernández-Vega, Fundación de Investigación Oftalmológica & Universidad de Oviedo, 33012 Oviedo, Spain; carlotasb.8@gmail.com (C.S.-B.); evagp2002@gmail.com (E.G.-P.); enol.artime@fio.as (E.A.); bbaamonde@yahoo.es (B.B.-A.); prof.luis@fernandez-vega.com (L.F.-V.); merayo@fio.as (J.M.-L.); 2 Instituto de Investigación Sanitaria del Principado de Asturias, Avenida de Roma s/n, 33011 Oviedo, Spain; 3 BTI Biotechnology Institute, 01007 Vitoria, Spain; maria.delafuente@bti-implant.es (M.d.l.F.); francisco.muruzabal@bti-implant.es (F.M.); dentalresearch@fundacioneduardoanitua.org (E.A.); 4 University Institute for Regenerative Medicine and Oral Implantology—UIRMI (UPV/EHU-Fundación Eduardo Anitua), 01007 Vitoria, Spain

**Keywords:** retina, plasma rich in growth factors (PRGF), blue light, degeneration, electrical signaling, photoreceptor, retinal injury

## Abstract

Purpose: The purpose of this study was to examine the effect of plasma rich in growth factors (PRGFs) under blue light conditions in an in vivo model of retinal degeneration. Methods: Male Wistar rats were exposed to dark/blue light conditions for 9 days. On day 7, right eyes were injected with saline and left eyes with PRGF. Electroretinography (ERG) and intraocular pressure (IoP) measurements were performed before and after the experiment. After sacrifice, retinal samples were collected. Hematoxylin and eosin staining was performed to analyze the structure of retinal sections. Immunofluorescence for brain-specific homeobox/POU domain protein 3A (Brn3a), choline acetyltransferase (ChAT), rhodopsin, heme oxygenase-1 (HO-1), and glial fibrillary acidic protein (GFAP) was performed to study the retinal conditions. Results: Retinal signaling measured by ERG was reduced by blue light and recovered with PRGF; however, IoP measurements did not show significant differences among treatments. Blue light reduced the expression for Brn3a, ChAT, and rhodopsin. Treatment with PRGF showed a recovery in their expressions. HO-1 and GFAP results showed that blue light increased their expression but the use of PRGF reduced the effect of light. Conclusions: Blue light causes retinal degeneration. PRGF mitigated the injury, restoring the functionality of these cells and maintaining the tissue integrity.

## 1. Introduction

The role of artificial light in retinal degeneration has been gaining attention as a possible risk factor [1,2,3,4,5,6,7]. White light, which corresponds to visible light, is commonly formed by different fractions of short wavelengths, including the blue light spectrum. This short wavelength light reaches the retinal cells, causing a disruption in their molecular bases and finally damaging the cell structure [3,8,9,10]. The presence of risk factors, such as obesity, smoking, and aging, on top of pre-existing eye pathologies, can lead to a progressive decline in retinal cells’ integrity and, eventually, loss of vision [11,12,13,14,15,16].

Specifically, blue light is known to increase the presence of reactive oxygen species (ROS). These molecules involve a deregulation of the main enzymes of the retinal metabolism present in the mitochondrial electron transport chain, producing a progressive interruption of ATP production, thereby promoting cell death. This can lead to a decrease in the number of visual cells, inducing a reduction in their functionality and, consequently, loss of vision. These cells are photoreceptors, such as cones and rods, and ganglion cells, amongst others [17,18,19,20,21].

Researchers have been attempting to find a solution to this problem for several years. For instance, plasma rich in growth factors (PRGF), which is a serum obtained from a patient’s blood, has been already proven to enhance cell proliferation, help with wound healing, and act as a neuroprotector or an anti-inflammatory agent [22,23,24]. It has been widely used in oral implantology and traumatology, and in ophthalmology for treating ocular surface diseases, such as persistent epithelial defects (PEDs) [25,26,27,28,29], conjunctivitis, or other ocular surface pathologies [30,31]. In the retina, PRGF has been proven to reduce macular holes [32,33]. Our group previously tested its effect on retinal pigment epithelium (RPE) through the reduction in the harm produced by oxidative stress. It was found to decrease the effect of blue light by activating the different antioxidant pathways while blocking its damage to cells [34,35].

Therefore, the aim of this work was to check if PRGF can also protect the visual function in situ by preserving neuronal cells in the retina, such as ganglion cells and photoreceptors, against the action of damaging insults from blue light.

## 2. Results

### 2.1. Electroretinogram and Intraocular Pressure (IoP)

**Figure 1:**
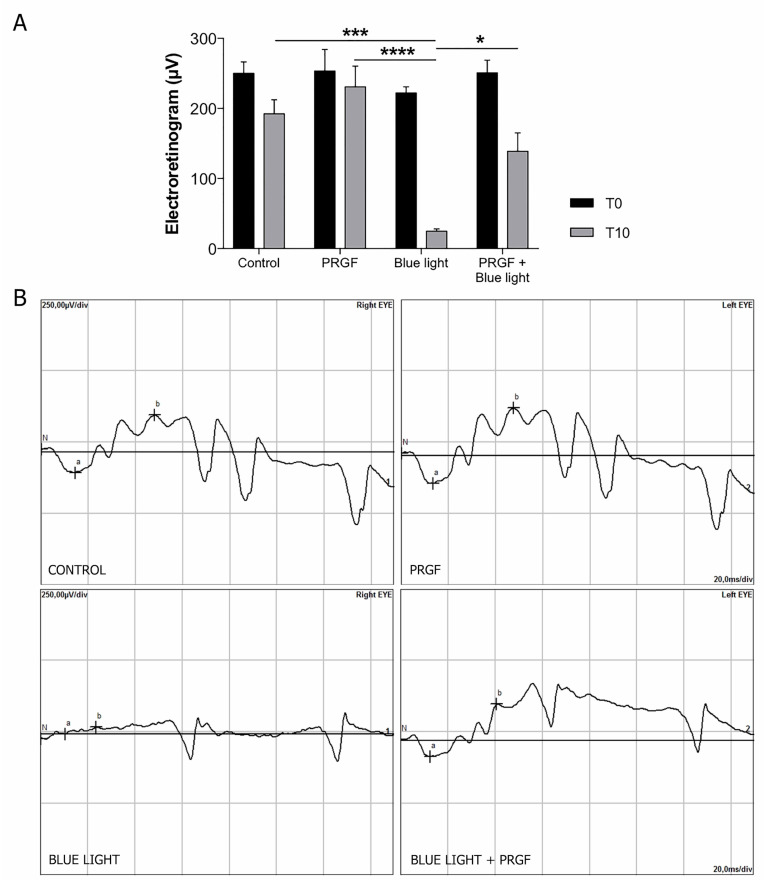
(**A**) Graphical representation of wave b measurements obtained from electroretinography (ERG) in scotopic 3.0 conditions at time 0 and time 10 (*n* = 4). T10 results showed that blue light reduced significantly the electrical signal produced by rat retinas compared to control. However, plasma rich in growth factors (PRGF) combined with blue light recovered the signaling pattern. We also found significant differences in the interaction between time and treatment (*p* = 0.036). Simple statistical analysis for time showed significant differences (*p* < 0.0001) probably produced by the experimental procedure. The treatment parameter also showed significant differences (*p* = 0.0001), indicating that blue light reduced the electrical signal and PRGF recovered it. Statistical analysis: Two-way ANOVA, Tukey’s multiple comparison test; * *p* < 0.05, *** *p* < 0.0005, **** *p* < 0.0001. (**B**) ERG diagram at time 10. Control and PRGF-treated retinas showed a normal electrical signal. Retinas treated with blue light showed a disruption of the electrical signal, which was recovered close to control values when blue light was combined with PRGF.

ERG results showed that the electrical signal at day 10 was lower compared to those obtained on day 0 in every group except for PRGF, which had a similar response at both timepoints. This decrease may have been caused by the injection, which may have damaged the cells. However, PRGF mitigated this effect. On day 10, blue light disrupted the signaling pattern compared to the control. Nevertheless, when blue light was combined with PRGF, the electrical signal started recovering closer to the control results (Figure 1).

**Figure 2:**
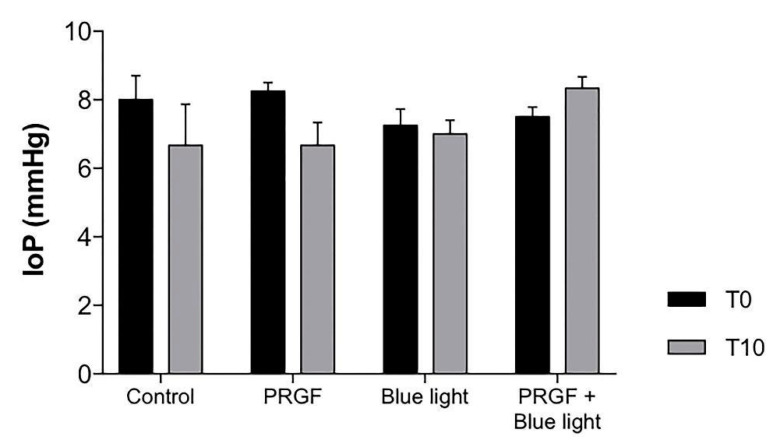
Intraocular pressure (IoP) values on day 0 (T0) and day 10 (T10) (*n* = 4). On day 0, the IoPs from each group were similar (7–8 mmHg). On day 10, IoP values of control, blue light, and PRGF treatments decreased to a mean value of 6–7 mmHg. However, PRGF combined with blue light kept the intraocular pressure closer to basal values. Statistical analysis: Two-way ANOVA, Tukey’s multiple comparison test.

The IoPs of animals were controlled during the experiment. The intraocular injection of saline or PRGF increased the IoP and produced an undesirable effect in the retina. The results showed that after 10 days, mean IoP values decreased to 6–7 mmHg compared to day 0 results (Figure 2). This result suggested that the injection could cause a leak in vitreous humour but did not show a potential additional insult to the retina.

### 2.2. Retinal Degeneration Can Involve Changes in Retinal Thickness

**Figure 3:**
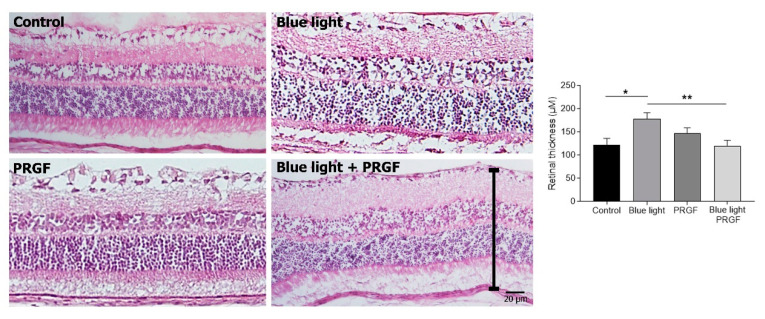
Hematoxylin and eosin (H&E) staining of transversal sections of experimental retinas (*n* = 4). PRGF combined with blue light maintained the tissue close to control conditions. However, blue light disrupted the retina’s integrity. Means of quantification thickness showed that there were significant differences between the result obtained from blue-light-exposed retinas and those treated with a combination of blue light and PRGF. Statistical analysis: One-way ANOVA, Sidak’s multiple comparison test, * *p* < 0.05 and ** *p* < 0.005. Scale = 20 µm.

We also studied the effect of PRGF and blue light on retinal thickness. The retinas of the four experimental groups stained with H&E were analyzed using ImageJ software to quantify the length from ganglion cells layer to the RPE by taking five different measurements along the retina, and means were calculated. The results showed that blue light caused damage to the retina, increasing its thickness by creating holes amongst cells and conferring a relaxed structure (Figure 3). When blue light was combined with PRGF, the retina maintained its integrity compared to the control.

### 2.3. Immunofluorescence Study

To check the integrity and functionality of the retina after blue light exposure, we studied the action of three different markers: brain-specific homeobox/POU domain protein 3A (Brn3a), which is a ganglion cell marker; choline-acetyltransferase (ChAT), which is a marker for cholinergic amacrine cells; and rhodopsin, which is located in rods and plays a role in visual function.

**Figure 4:**
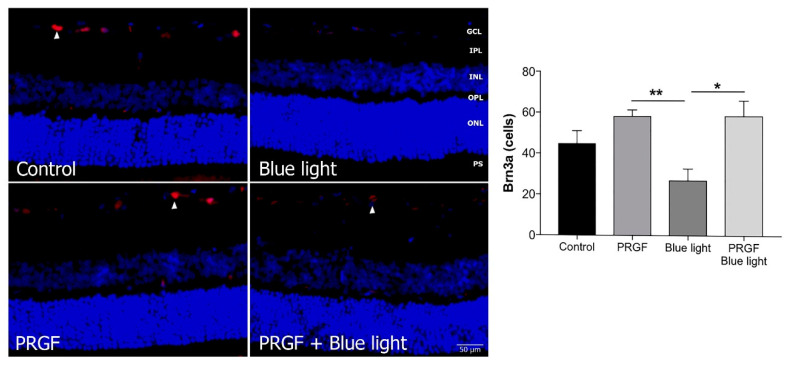
Immunofluorescence for Brn3a (red) and DAPI (blue) in transversal sections of retinas (*n* = 4). The results showed that the expression of Brn3a was interrupted by blue light compared to control and PRGF treatment conditions. However, when blue light was combined with PRGF, its expression recovered. Arrowheads show positive cells for Brn3a. Statistical analysis: One-way ANOVA, Sidak’s multiple comparison test (*n* = 3), * *p* < 0.05 and ** *p* < 0.05. Scale = 50 µm.

Immunofluorescence for Brn3a (Figure 4) showed that blue light decreased the number of positive cells compared to the control and PRGF conditions. Brn3a positive cells were quantified by using ImageJ software. For that, we selected sections from the whole retina and positive cells were counted. Analysis indicated that PRGF recovered the damage caused by blue light. These results also suggest that PRGF reduced the damage caused by the injection to ganglion cells, as the PRGF treatment’s count showed a higher number than the control. When the light was combined with PRGF, the expression of this protein in ganglion cells recovered to control conditions.

**Figure 5:**
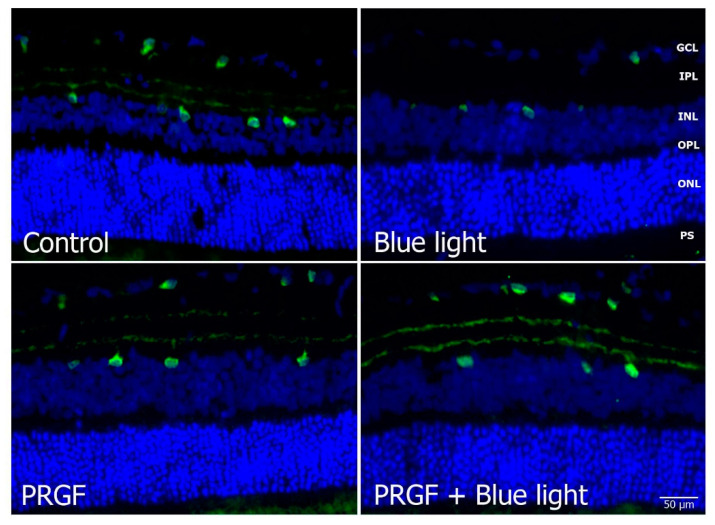
Immunofluorescence for ChAT (green) and DAPI (blue) in transversal section of retinas (*n* = 4). The results showed that blue light decreased its expression compared to the control. However, PRGF combined with blue light recovered the expression. Scale = 50 µm.

ChAT immunofluorescence results (Figure 5) showed that blue light decreased the expression of this enzyme, which is recuperated when it is combined with PRGF. PRGF alone showed an expression similar to control.

**Figure 6:**
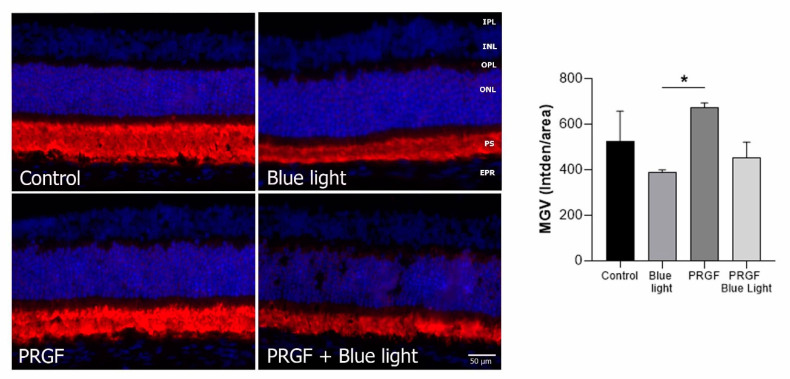
Immunofluorescence for rhodopsin (red) and DAPI (blue) in transversal section of retinas (*n* = 4). The results showed that the expression of rhodopsin was reduced by blue light compared to PRGF treatment conditions. Statistical analysis: One-way ANOVA, Sidak’s multiple comparison test, * *p* < 0.05. Scale = 50 µm.

To analyze the differences within rhodopsin results, we also quantified the fluorescence using ImageJ software. The software compares the intensity of the expression related to the thickness of the tissue, or mean grey value (MGV). We found (Figure 6) that blue light significantly reduced the rhodopsin expression compared to PRGF treatment. Regarding the combination of PRGF and blue light, there were no statistically significant differences. However, we observed a positive tendency toward control results.

To assess oxidative stress and retinal damage in our samples, the expressions of heme oxygenase-1 (HO-1) and glial fibrillary acidic protein (GFAP), respectively, were studied.

**Figure 7:**
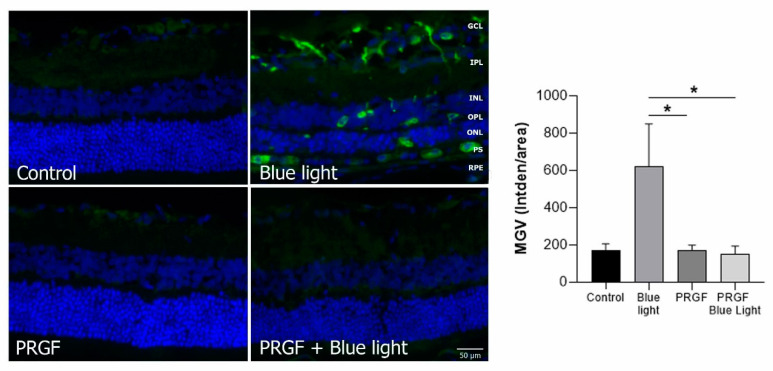
Immunofluorescence for HO-1 (green) and DAPI (blue) in transversal section of retinas (*n* = 4). The results showed that the expression of HO-1 was increased by blue light compared to PRGF treatment conditions. Differences between control and blue light were also detected; however, statistical analysis did not show significance (*p* = 0.0578). Statistical analysis: one-way ANOVA, * *p* < 0.05. Scale = 50 µm.

For analyzing HO-1, we also studied the MGV. The results (Figure 7) showed that blue light significantly increased the expression of HO-1, and this was mitigated by PRGF. PRGF, alone or in combination with blue light, did not show expression for this marker. This result suggested that blue light increases the expression of oxidant markers such as HO-1 and that PRGF reduces it.

**Figure 8:**
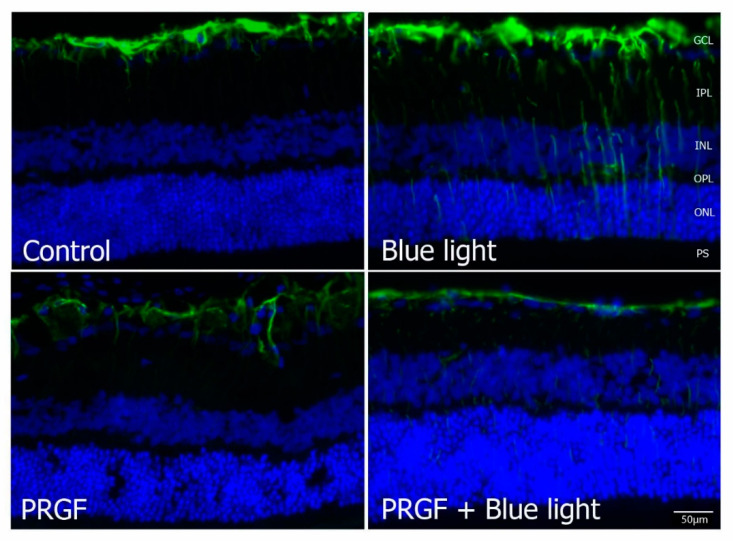
Immunofluorescence for GFAP (green) and DAPI (blue) in transversal section of retinas (*n* = 4). The results showed that blue light increased its expression compared to control. However, PRGF combined with blue light reduced the expression, even more than that in the control results. Scale = 50 µm.

Immunofluorescence for GFAP (Figure 8) showed that blue light also increased the expression of this marker, which decreased in the presence of PRGF. This result suggested that blue light increased the damage to retinal cells, activating the response of glial cells and producing scarring, and PRGF protected them.

## 3. Discussion

The retina is one of the most important parts in the eye, due to the role it played in visual function. This tissue is composed of different cell types, such as neuronal cells, photoreceptors, and epithelial cells, which are involved in the biochemical processes that are carried out in this tissue. For instance, photoreceptors oversee the process of sending electrical stimuli through the optical nerve to the brain to create images [36,37]. Epithelial cells are involved in the protection of and nutrient transport to photoreceptors [38]. As such, maintenance of retina integrity is essential [39].

Many factors can affect retinal cell survival, such as aging, obesity, or smoking. However, in the last few years, researchers have become more aware of light as a risk factor due to its presence in contemporary life. White light includes different short wavelengths, such as blue light. This type of light reaches the retinal cells and has the potential to disrupt their functions, thereby damaging the structure. This stimulus, combined with other factors such as previous pathologies, can lead to an ongoing degeneration and, finally, visual loss [4,5,8,9,13,20,40,41,42,43,44,45,46].

Researchers have been attempting to determine how to delay the degeneration process. In this regard, PRGF, which is a serum extracted from a patient’s blood, has been proven to be an enhancer of cell regeneration, a stimulator of wound healing, a neuroprotector, and an anti-inflammatory agent [22,23,24,47,48]. It has been used in oral implantology and traumatology. In ophthalmology, it has been used for treating eye surface disorders such as PED or dry eye [25,27,28,29,31,47,49]. In terms of pathologies related to the retina, PRGF has also been used to treat macular holes, producing good results in both anatomical closure and vision recovery [32,33]. Other experimental studies demonstrated the neuroprotector role of PRGF in the retina. We previously studied the PRGF antioxidant effect, reverting the damage produced after blue light exposure and enhancing cell survival in RPE cells [34,35]. Oxidative stress is involved in the pathophysiology of retinal degenerations, such as age-related macular degeneration (AMD) and glaucoma, and exogenous factors, such as short wavelength light, can exacerbate those diseases.

ERG measurements are useful for studying the retinal state, as they indicate the response to a luminous stimulus. The study of scotopic conditions enables the analysis of the functional state of photoreceptors. The results indicated that at day 10, the electrical signal was lower compared to day 0 in all the treatments except for PRGF. This reduction might have been caused by the injection, which may have damaged cells and produced a slight leak in the vitreous humour, as evidenced by the IoP measurements. When examining the results obtained on day 10, blue light significantly reduced the electrical signal produced by retinal cells. This was mitigated when the eye was treated with PRGF, showing similar results to the control. This suggests that PRGF reduces the harm produced by blue light in terms of functionality. PRGF did not produce an increase in IoP. Notably, the administration of PRGF did not cause an increase in IoP, as it could be derived from the secondary effects, similar to those in diseases such as glaucoma.

The maintenance of the integrity of retinal structure is essential for preserving visual function. The analysis of retinal transversal sections showed that blue light significantly increased the thickness of the retina due to the disruption of tissue integrity. However, PRGF preserved the retinal structure even when light exposure was applied. Some authors suggested that light can disrupt the cellular structure of the retina. First, photoreceptors become swollen and tortuous. After a few days of light exposure, the mitochondria of the anterior segment become swollen too and chromatin condenses [8,50,51].

PRGF was demonstrated to be useful for the protection of several cell types. Brn3a is a marker of retinal ganglion cells [52,53,54]. Blue light produced a loss in positive cells for Brn3a, which was reverted when PRGF was injected. This corresponds to the observations reported by other authors, which showed that blue light reduces the survival of ganglion cells [51], reducing the collection of visual information that is sent to photoreceptors and, therefore, the visual function. Similar results were obtained in the study of ChAT, which is a marker for neurons [55,56]. This investigation showed that its expression lowered in the presence of blue light but increased to control levels when combined with PRGF. The reduction in the expression of this marker showed that the electrical signal may not be sent through these cells to photoreceptors, causing a reduction in visual function. Rhodopsin, which is a rod marker [14,15,57,58,59], staining showed that blue light reduced the thickness of the rod layer and the fluorescence intensity. In this regard, the reduction in rhodopsin produced by blue light contributed to the deterioration in vision, as rods are involved in visual function under dark conditions. GFAP is expressed by several cells of the central nervous system cells, such as astrocytes and Müller cells, after exposure to an insult. It is also related to other structural proteins such as vimentin, and it is responsible for creating scars by interacting with the fibrous tissue. Therefore, it is a suitable marker for glial damage [60,61,62,63,64]. Immunofluorescence results for this protein showed that blue light increased the glial damage, which was reverted when light was combined with PRGF. All results suggested that blue light reduces the functionality of retinal cells and treatment with PRGF reduces the impact of the insult even when the treatment is administered once the damage is initiated.

Blue light produces oxidative stress, which can be detected by the increase in HO-1 expression [44,65,66,67,68]. The antioxidant capacity of PRGF reduced the expression of HO-1 to control levels. Our previous work regarding oxidative damage caused by blue light proved that blue light increases the presence of ROS, thereby enhancing HO-1 staining [35]. In the present study, the HO-1 staining results also showed that blue light significantly increased the expression of this protein, suggesting a high presence of oxidant products. This was reduced to control results when PRGF was present, suggesting that PRGF blocks the oxidant pathway, protecting cells against oxidative stress.

As the study limitations, we found that the injections of both saline and PRGF could damage retinal cells, thereby complicating the analysis of the different responses to the treatments. The study only included one PRGF injection in a short period of time and the time that PRGF can protect cells remains unknown. Nevertheless, the data suggest that PRGF reduced the damage caused by the invasive experimental procedure. However, future investigations are needed to test the efficiency of PRGF in the retina for a longer duration.

Finally, we concluded that PRGF enhances the integrity and retinal function when there is oxidative damage caused by exposure to blue light. Future research work is needed to deepen our understanding of how PRGF protects cells and its potential therapeutic use in retinal degeneration.

## 4. Animals, Materials and Methods

### 4.1. PRGF

In accordance with the Declaration of Helsinki of 2013, blood from four different healthy donors (all women, mean age 33 ± 7 years) was collected and placed in 9-mL tubes with 3.8% sodium citrate (Vacuette tube, Greiner Bio-One, Kremsmünster, Austria). The blood was then centrifuged at room temperature (Endoret System, BTI Biotechnology Institute, S.L., Vitoria, Spain). Whole-plasma was collected after centrifugation, avoiding the leukocyte layer, and transferred to a 15-mL tube. Plasma was mixed with calcium chloride for fibrinogen activation and incubated for 30 min at 37 °C, or until clotting was achieved. The supernatant was collected and exposed to heat (56 °C) for 1 h to inactivate the complement system. After that, the plasma was filtered, aliquoted, and kept at −4 °C until use (less than 6 months).

### 4.2. Animals

This study was performed in accordance with the association for research in vision and ophthalmology (ARVO) Statement for the Use of Animals in Ophthalmic and Vision Research. The procedures and experimental designs were approved by the Animal Experimentation Ethics Committee of the University of Oviedo (Oviedo, Principado de Asturias, Spain; PROAE 17/2017, approved on 23/03/2017) and complied with European and national laws.

**Figure 9:**
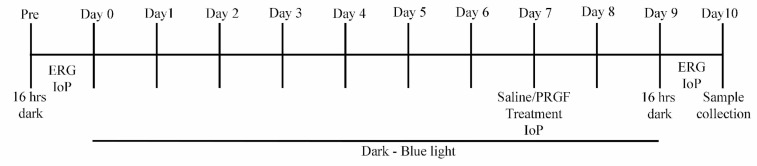
Experimental design diagram.

**Figure 10:**
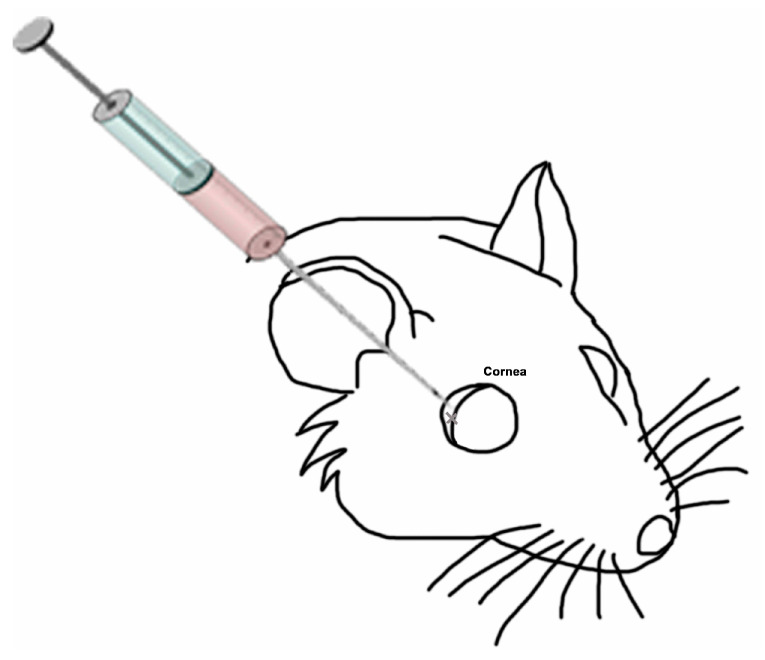
Diagram of eye injection.

Wistar male rats weighing about 500 g were divided into dark and blue light groups (Table 1) and treated by the following experimental design (Figure 9): the day prior to the experiment, animals were moved and kept in the dark for 16 h. They were anaesthetized with ketamine/xylazine 80/10 mg/kg. A second injection of 1/3 of the initial anesthetic solution was used to keep the rats asleep for 60 min. After that, electroretinogram (ERG) and intraocular pressure (IoP) measurements were performed. For 7 days, animals were exposed either to dark or blue light depending on the experimental group. Blue light LEDs (Electro DH, SL, Barcelona, Spain) were used to deliver light to the rat eyes at 465–475 nm (10 W/m^2^) for 4 h each day. On day 7, right eyes were injected with 10 µL of saline solution and left eyes with 10 µL of PRGF using a Hamilton syringe in the sclera, right after the corneal limbus (Figure 10). IoP was also measured to observe differences. After this procedure, animals were returned to their experimental dark/light exposure pattern. On day 9, animals were moved and kept in dark conditions for 16 h. After that time and after being anaesthetized, ERG and IoP measurements were performed. Finally, animals were euthanized with a pentobarbital injection and their eyes enucleated. To conclude, eyes were fixed with paraformaldehyde 4% for 2 h and frozen in optimal cutting temperature (OCT) compound.

### 4.3. Electroretinogram and Intraocular Pressure

Animals were exposed to the ERG electrophysiology assay (RETIAnimal, Roland Consult, Brandenburg, Germany) to analyze the retinal state before and after dark/blue light experimental treatment. First, animals were anaesthetized as previously described and located in the device. After applying a Gonioftal (Alcon Healthcare, S.A. Barcelona, Spain) drop into both corneas, electrodes were located. A ground was also located on the base of the tail, with one reference electrode on each scapula and the activated electrodes in both corneas. Light stimulus was generated with a white LED (–30 dB, 0.003 cd/m^2^, and 0.125 Hz). Measurements were recorded under scotopic conditions and animals were adapted to darkness for 16 h.

Intraocular pressure was measured with a Tonolab tonometer (Icare Tonolab, Vantaa, Finland) on days 0 and 10.

### 4.4. Immunofluorescence

The eyes of eight male Wistar rats were fixed in cold paraformaldehyde 4% for 2 h and cut in transversal sections by cryostat Microm HM 550 (Thermo Scientific, Waltham, MA, USA). Then, they were washed in phosphate buffered saline (PBS). After incubation in 10% goat serum (Vector Laboratories, Burlingame, CA, USA) or donkey serum (Jackson InmunoResearch Europe Ltd., Ely, Cambridgeshire, U.K.) in PBS for 60 min and washing in PBS, the retinal sections were then exposed overnight at 4 °C to primary antibodies (Table 2). After washing with PBS, tissues were then exposed for 2 h to the appropriate secondary antibody conjugated with either Alexa Fluor 488 or Alexa Fluor 594 (1:300) and washed in buffer. In the end, DAPI (4′,6-diamidino-2-phenylindole) (0.2 μg/mL) was added to a wash solution. Images were obtained using a Leica DMI6000B fluorescence microscope (Leica Microsystems, Wetzlar, Germany).

### 4.5. Histological Studies and Retinal Thickness Quantification

For histological studies, samples were dyed following the hematoxylin and eosin (H&E) regular protocol. Briefly, eye sections were exposed to hematoxylin for 3 min, rinsed with water, exposed to eosin for 2 min, and washed with distilled water for 1 min. After this, they were exposed to different concentrations of alcohol (70%, 80%, 96%, and 100%) for 20 s each, and xylene I and xylene II for 30 s each.

Images were obtained using a Leica DMI6000B fluorescence microscope (Leica Microsystems, Wetzlar, Germany).

Once pictures of H&E-stained sections were taken, images were processed in Fiji (ImageJ, National Institutes of Health, Bethesda, MD, USA). The thicknesses of retinas were quantified in five different areas for each group, and mean values were calculated.

### 4.6. Statistical Analysis

All statistical tests were analysed using GraphPad Prism version 7.0a for Mac (GraphPad Software, La Jolla, CA, USA). To assess the statistical significance, one-way and two-way ANOVAs were performed. For the statistical comparison of mean differences between treatments, we used Tuckey’s multiple comparison test and Sidak’s multiple comparison test. Differences were considered statistically significant when *p*-values were <0.05.

## Figures and Tables

**Table 1:** Experimental groups.

Treatment	Medium	Dark/Blue Light
**Control**	10 µL saline solution	Dark
**Blue light**	10 µL Saline solution	Blue light
**PRGF**	10 µL PRGF 100%	Dark
**Blue light + PRGF**	10 µL PRGF 100%	Blue light

**Table 2:** Antibodies used in this study.

Antibody	Reference (RRID)	Species	Dilution	Company
Primary Antibodies				
**HO-1**	Enzo Life Sciences Cat# SPA-894F, RRID:AB_991588	Rabbit	1:100	Enzo LS, Farmingdale, NY, USA
**GFAP**	Agilent Cat# Z0334, RRID:AB_10013382	Rabbit	1:500	Dako, Santa Clara, CA, USA
**ChAT**	Millipore Cat# AB144P, RRID:AB_2079751	Goat	1:250	Millipore, Burlington, MA, USA
**Brn3a**	Santa Cruz Biotechnology Cat# sc-31984, RRID:AB_2167511	Mouse	1:200	Santa Cruz, Dallas, TX, USA
**Rhodopsin**	Millipore Cat# MABN15, RRID:AB_10807045	Mouse	1:200	Millipore, Burlington, MA, USA
**Secondary Antibodies**				
**Anti-rabbit** **Alexa Fluor 488**	Thermo Fisher Scientific Cat# A32731TR, RRID:AB_2866491	Goat	1:300	ThermoFisher, Waltham, MA, USA
**Anti-goat Alexa Fluor 488**	Thermo Fisher Scientific Cat# A32814TR, RRID:AB_2866497	Donkey	1:300	ThermoFisher, Waltham, MA, USA
**Anti-mouse** **Alexa Fluor 594**	Thermo Fisher Scientific Cat# A32742, RRID:AB_2762825	Goat	1:300	ThermoFisher, Waltham, MA, USA
